# Molecular mechanism of nutrient uptake in developing embryos of oviparous cloudy catshark (*Scyliorhinus torazame*)

**DOI:** 10.1371/journal.pone.0265428

**Published:** 2022-03-15

**Authors:** Yuki Honda, Nobuhiro Ogawa, Marty Kwok-Shing Wong, Kotaro Tokunaga, Shigehiro Kuraku, Susumu Hyodo, Wataru Takagi

**Affiliations:** 1 Laboratory of physiology, Atmosphere and Ocean Research Institute, The University of Tokyo, Kashiwa, Chiba, Japan; 2 Atmosphere and Ocean Research Institute, The University of Tokyo, Kashiwa, Chiba, Japan; 3 Ibaraki Prefectural Oarai Aquarium, Oarai, Ibaraki, Japan; 4 Laboratory for Phyloinformatics, RIKEN Center for Biosystems Dynamics Research, Kobe, Japan; 5 Molecular Life History Laboratory, National Institute of Genetics, Mishima, Japan; 6 Department of Genetics, Sokendai (Graduate University for Advanced Studies), Mishima, Japan; University of Montreal, CANADA

## Abstract

Forms of embryonic nutrition are highly diverse in cartilaginous fishes, which contain oviparity, yolk-sac viviparity and several types of matrotrophic viviparity (histotrophy, oophagy, and placentotrophy). The molecular mechanisms of embryonic nutrition are poorly understood in these animals as few species are capable of reproducing in captivity. Oviparous cartilaginous fishes solely depend on yolk nutrients for their growth and development. In the present study, we compared the contribution to embryonic nutrition of the embryonic intestine with the yolk sac membrane (YSM). RNA-seq analysis was performed on the embryonic intestine and YSM of the oviparous cloudy catshark *Scyliorhinus torazame* to identify candidate genes involved in nutrient metabolism to further the understanding of nutrient utilization of developing embryos. RNA-seq discovery was subsequently confirmed by quantitative PCR analysis and we identified increases in several amino acid transporter genes (*slc3a1*, *slc6a19*, *slc3a2*, *slc7a7*) as well as genes involved in lipid absorption (*apob* and *mtp*) in the intestine after ‘pre-hatching’, which is a developmental event marked by an early opening of the egg case about 4 months before hatching. Although a reciprocal decrease in the nutritional role of YSM was expected after the intestine became functional, we observed similar increases in gene expression among amino acid transporters, lipid absorption molecules, and lysosomal cathepsins in the extraembryonic YSM in late developmental stages. Ultrastructure of the endodermal cells of YSM showed that yolk granules were incorporated by endocytosis, and the number of granules increased during development. Furthermore, the digestion of yolk granules in the YSM and nutrient transport through the basolateral membrane of the endodermal cells appeared to be enhanced after pre-hatching. These findings suggest that nutrient digestion and absorption is highly activated in both intestine and YSM after pre-hatching in catshark embryos, which supports the rapid growth at late developmental stages.

## Introduction

Cartilaginous fish possess diverse forms of reproduction from oviparity to placental viviparity [1,2]. In placentotrophic viviparous species, embryonic nutrients are mainly supplied through the blood circulation as in mammals [3]. Meanwhile, embryos of all oviparous and other viviparous species (yolk-sac, histotrophic, and oophagous) are known to be nourished by the yolk initially [4,5], and yolk is the sole nutrient source throughout the development in oviparous species. Thus, how the embryos partition the limited resource at different stages is a particularly important subject in development of oviparous species [6]. However, few studies have addressed the mechanisms underlying embryonic nutrition in cartilaginous fishes [7–9].

**Fig 1:**
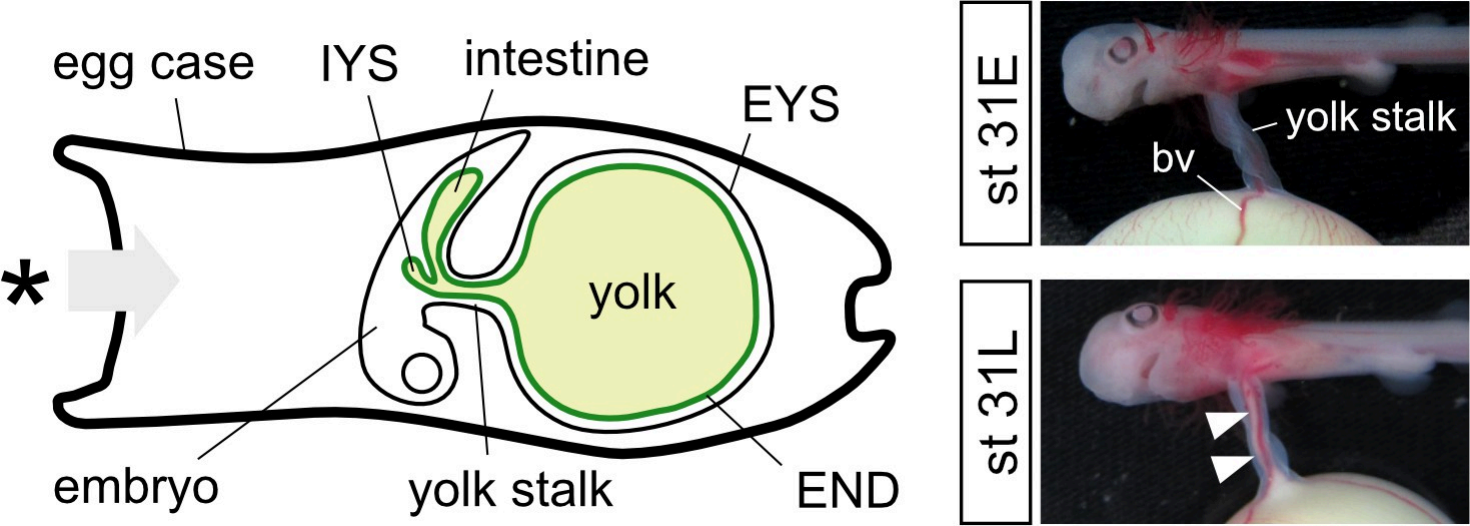
‘Pre-hatching’ in cloudy catshark. Schematic drawing and pictures of developing catshark embryo around pre-hatching period. The external yolk sac is attached to an embryo via the yolk stalk. Yolk contents inside the external yolk sac start to be transferred into the internal yolk sac and embryonic intestine through the stalk at stage 31L (arrowheads), but not in the embryo at stage 31E (before pre-hatching). Asterisk indicates the site where egg case opens during pre-hatching period. EYS, external yolk sac; IYS, internal yolk sac.

“Pre-hatching” is a developmental event experienced by oviparous cartilaginous fishes. It is characterized by an early opening of the egg capsule after approximately one-third of development [10–12]. Our previous findings in the cloudy catshark (*Scyliorhinus torazame*) demonstrated that the embryonic intestine develops the spiral valves and expressed several transporters for amino acids and small peptides at the pre-hatching period [13], suggesting that the intestine becomes functional among pre-hatched individuals. The developing embryo and two cellular layers of the external yolk sac (EYS) are connected via the yolk stalk. Yolk starts to be transferred from EYS to the embryonic intestine and internal yolk sac through the stalk just after pre-hatching (Fig 1) [13]. By the time of hatching, yolk contents are almost completely transferred into the embryo. In another oviparous species, the lesser spotted dogfish (*S*. *canicula*), transfer of yolk contents from the EYS to the embryonic intestine is also known to occur around the pre-hatching stage [7,10]. The embryonic intestine is thus assumed to be the major site for embryonic nutrition in the oviparous elasmobranchs after the pre-hatching event.

In contrast, how the oviparous cartilaginous fish embryos utilize the yolk before pre-hatching remains debatable. The yolk sac membrane (YSM) is a major site for digestion and absorption of nutrients in early development of spiny dogfish *Squalus acanthias*, since active degradation of yolk droplets in lysosomes was observed only in the YSM of early developmental stages (less than 90 mm in body length) [14,15]. It was also reported that yolk platelets are degraded in the yolk syncytial layer and the resulting yolk granules are incorporated into endodermal cells by endocytosis before implantation in viviparous carcharhinid sharks [16,17]. However, Lechenault et al. [7] demonstrated that endodermal epithelial cells of the YSM in oviparous *S*.*canicula* become activated only when the EYS begins to shrink, which happens just after pre-hatching. To date, no molecular-based evidence is known to support the claim that yolk nutrients are transported into the vitelline circulation from the endodermal cells of the YSM.

Several studies on other vertebrates suggest the importance of the YSM for embryonic nutrition. In chicken embryos, the uptake of yolk nutrients is mediated by a variety of amino acid and monosaccharide transporters expressed in the intestine and YSM [18–20]. Several digestive enzyme genes such as cathepsin A (ctsa) and B (ctsb) were also highly expressed in the intestine and YSM during chicken development, which likely digest the large yolk molecules to small molecules to be absorbed by transporters [21]. Since proteins and lipids occupy a relatively large extent of the yolk contents in cartilaginous fishes [22,23], the reported amino acid transporters and digestive enzymes can be candidates for the understanding of the absorption mechanisms of yolk nutrients.

Here, we investigated the molecular and histological aspects of YSM with focus on digestion and absorption function in the cloudy catshark, and compared the results to those of the intestine throughout embryonic development. We found upregulation of many amino acid transporters and lipid absorption proteins in the embryonic intestine after pre-hatching, in accord with our previous study [13]. The expression of some important amino acid transporters, lipid transport proteins and lysosomal digestive enzymes were also prominent in the YSM after pre-hatching, suggesting that the catshark embryo metabolize the yolk by both YSM and intestine to maximize the nutrient absorption capacity before hatching.

## Materials and methods

### Animals and ethics statement

Embryos and juveniles of catshark were maintained and sampled as described in the previous study [13]. Fertilized eggs collected from captive individuals were transported from Ibaraki Prefectural Oarai Aquarium to the Atmosphere and Ocean Research Institute at the University of Tokyo. They were kept in a 1000-liter tank with recirculating natural seawater under a constant photoperiod (12 h:12 h light:dark) at 16°C. After the anesthesia with MS-222 (0.02% in seawater), the embryo and the EYS were separated, and the wet weight was measured. The developmental stages of cloudy catshark embryos were identified according to the previous study [13]. The embryo and YSM were then snap frozen or fixed with modified Bouin’s fixative (formalin:saturated picric acid = 1:3) for further genetic and histological analyses, respectively. All animal experiments were conducted according to the Guidelines for Care and Use of Animals approved by the committee of The University of Tokyo (A16-13).

### RNA-seq analysis

RNA-seq analysis was performed on the embryonic intestine and YSM. The YSM of catshark can be divided into two layers: a highly vascularized inner layer (YSMin), and an ectodermal outer layer (YSMout). Two YSM layers (YSMin and YSMout) were sampled from embryos of stages 31E (before pre-hatching; *N* = 3) and 32 (after pre-hatching; *N* = 3). The intestines were sampled from stage 28 (*N* = 3) and stage 32 (*N* = 3) embryos. Total RNA was extracted from samples using ISOGEN (Nippon Gene, Toyama, Japan), treated with DNase I, and purified with RNA Clean & Concentrator (Zymo Research, CA, USA). The concentration and quality of the extracted RNA were assessed using a Qubit 2.0 Fluorometer (Thermo Fisher Scientific, Waltham, MA, USA) and an Agilent 2100 Bioanalyzer (Agilent Technologies, Palo Alto, CA, USA), respectively. Libraries were prepared with 370 ng (intestine) or 1 μg (YSM) purified RNA using a TruSeq total RNA sample preparation kit (Illumina, San Diego, CA, USA). Sequencing was performed with HiSeq 1500 (Illumina) to obtain single end 80 bp reads [24]. The adaptor sequences and low-quality reads were discarded using Trim Galore! v0.3.1 (https://www.bioinformatics.babraham.ac.uk/projects/trim_galore/) and a fastq quality filter v0.10.0 (https://www.bioinformatics.babraham.ac.uk/projects/fastqc/).

*De novo* transcriptome assembly was performed to obtain transcript contigs using Trinity version 2.4.0 [25]. The contigs were annotated using ncbi-blast-2.6.0+ [26]. The trimmed sequence reads were mapped to the contigs using bowtie2 v2.3.2 [27] and the Transcripts Per Million (TPM) values were quantified using eXpress v1.5.1 [28].

### Candidate gene selection

The *de novo* assembled contigs of both YSM and embryonic intestine were annotated after BLASTX search, and the candidate genes involved in amino acid and lipid absorption were identified. For amino acid transporters, the candidate genes were selected mostly based on the findings from mammalian intestines (Table 1) [29]. Although *slc6a18* is mainly expressed in proximal tubule of the nephron in mammals [30], its orthologue in *Mozambique tilapia* is primarily expressed in the intestine and likely responsible for amino acid absorption through the intestinal epithelia [31]. Thus, we included *slc6a18* as a candidate to be examined in the present study. The putative transport system and substrates of examined transporters were summarized in Table 1 based on mammalian studies [30,32–36]. The genes involved in lipid absorption were selected based on studies on the mammalian intestine [37]. Apolipoprotein B (ApoB) is a major component of chylomicron and the microsomal triglyceride-transfer protein (MTP) is essential for assembly and secretion of apoB-containing lipoproteins [38]. Apolipoprotein A1 (ApoA1) is a major component of high-density lipoprotein (HDL) and ATP-binding cassette subfamily A member 1 (ABCA1) is known to promote lipidation of ApoA1 to form HDL (Table 2) [39]. To evaluate protein digestion capacity in the YSM, putative cathepsin genes were identified by screening the transcriptome data with a cut-off TPM value >100. Four cathepsin genes were identified: cathepsin L1 (*ctsl1*), cathepsin L2 (*ctsl2*), cathepsin F1 (*ctsf1*), cathepsin F2 (*ctsf2*) (Table 2). In addition, *ctsa* and *ctsb* were also examined, since they are involved in the yolk protein digestion in chicken [19]. For lipid digestion, a lysosomal acid lipase (LIPA) gene was also selected in this study [19] (Table 2).

**Table 1:** Amino acid transporter genes expressed in yolk sac membrane and intestine identified in transcriptome analysis.

Putative localization	Transcript contig ID	Gene name	Protein name	Putative transport type	Putative substrates	TPM values		
						31E YSMin	32 YSMin	32 Intestine
Apical	TRINITY_DN58341_c0_g1	*slc15a1*	PEPT1	C/H+	di- and tri-peptides, protons, beta-lactam antibiotics	0.01	0.03	32.76
Apical	TRINITY_DN68157_c9_g2	*slc3a1*	rBAT	E	heterodimerizes with light subunit SLC7A9	3.72	0.25	10.40
	TRINITY_DN35499_c0_g1	*slc6a18*	SLC6A18		neutral amino acids	2.73	0.45	0.58
	TRINITY_DN60712_c0_g1	*slc6a19*	SLC6A19		neutral amino acids	0.12	0.17	45.51
	TRINITY_DN61159_c0_g1	*slc6a20*	SLC6A20		P, pipecolate, sarcosine	1.16	4.59	6.20
	TRINITY_DN66656_c0_g1	*slc7a9*	b0,+AT	E (preferentially extracellular cationic amino acid and cystine against intracellular neutral amino acid)	cationic amino acids, large neutral amino acids	20.51	30.83	14.49
	TRINITY_DN143119_c0_g1	*slc36a1*	PAT1	C/H+	GABA, P, G, beta-A	0.37	2.08	0.57
Basolateral	TRINITY_DN67547_c1_g1	*slc3a2*	4F2hc	E	associated with light subunits SLC7A5-8 and 10–11	44.99	39.72	71.33
	TRINITY_DN68243_c0_g1	*slc7a6*	y+LAT2	E (preferentially intracellular cationic amino acid against extracellular neutral amino acid/Na+)	cationic amino acids (Na+ independent), large neutral L-amino acids (Na+ dependent)	0.66	1.20	2.67
	TRINITY_DN70063_c17_g1	*slc7a7*	Y+LAT1	E (preferentially intracellular cationic amino acid against extracellular neutral amino acid/Na+)	cationic amino acids (Na+ independent), large neutral L-amino acids (Na+ dependent)	0.23	0.42	14.82
	TRINITY_DN57982_c0_g1	*slc7a8*	LAT2	E (similar intra- and extracellular selectivities, lower intracellular apparent affinity)	neutral L-amino acids, T3, T4, BCH	13.53	14.56	36.94
	TRINITY_DN58066_c0_g1	*slc38a2*	SNAT2	C/Na+	A, N, C, Q, G, H, M, P, S	3.75	2.49	4.12
	TRINITY_DN37134_c0_g1	*slc43a2*	LAT4	F	L-BCAAs, amino alcohols	1.03	18.60	7.51
Other	TRINITY_DN53217_c0_g1	*slc1a6*	EAAT4	C/Na+, H+, K+	L-E, D/L-D	1.26	1.58	0.52
	TRINITY_DN70444_c0_g1	*slc7a2*	CAT-2	F	cationic L-amino acids	31.37	50.50	0.10
	TRINITY_DN53940_c0_g1	*slc7a3*	CAT-3	F	cationic L-amino acids	3.51	5.33	3.24
	TRINITY_DN52522_c0_g1	*slc36a4*	PAT4	C/H+	P, W	0.54	1.73	2.96
	TRINITY_DN64338_c0_g1	*slc38a5*	SNAT5	C/Na+, E/H+	Q, N, H, S	0.33	1.09	0.65
	TRINITY_DN62704_c1_g1	*slc38a7*	SNAT7	?/Na+	Q. H, S, A, N	1.64	2.73	7.51

TPM, transcripts per kilobase million. Abbreviations for transport type: **C:** Cotransporter; **E:** Exchanger; **F:** Facilitated transporter.

**Table 2:** Lipid absorption genes, cathepsin, and LIPA genes expressed in yolk sac membrane and intestine identified in transcriptome analysis.

Putative function	Transcript contig ID	Gene name	Putative gene product	TPM values		
				31E YSMin	32 YSMin	32 Intestine
Lipid absorption	TRINITY_DN70098_c4_g1	*apob*	Apolipoprotein B	3089.08	4146.9	4838.06
	TRINITY_DN64661_c0_g1	*mtp*	Microsomal triglyceride transfer protein	5.77	9.73	35.97
	TRINITY_DN69420_c0_g2	*apoa1*	Apolipoprotein A-1	551.29	601.18	1678.74
	TRINITY_DN70132_c0_g1	*abca1*	ATP-binding cassette sub-family A member 1	11.00	8.41	34.92
Protein digestion	TRINITY_DN69767_c3_g3	*ctsl2*	Cathepsin L2	4496.82	3469.43	111.63
	TRINITY_DN68938_c0_g1	*ctsf1*	Cathepsin F1	450.02	97.06	24.16
	TRINITY_DN65741_c0_g1	*ctsl1*	Cathepsin L1	125.03	147.09	375.00
	TRINITY_DN68938_c0_g2	*ctsf2*	Cathepsin F2	164.92	38.07	14.17
	TRINITY_DN64326_c0_g1	*ctsb*	Cathepsin B	25.11	61.85	122.63
	TRINITY_DN69202_c20_g1	*ctsa*	Cathepsin A (Lysosomal protective protein)	7.62	12.98	19.36
Lipid digestion	TRINITY_DN52439_c0_g1	*lipa*	Lysosomal acid lipase	8.09	10.66	11.40

TPM, transcripts per kilobase million.

### cDNA cloning

Complementary DNA cloning was performed as previously described [13], using gene specific primer sets (S1 Table). The primer sets were designed based on the putative nucleotide sequences of mRNAs obtained from the *de novo* assembled fasta file, or BLAST searches on the sequence archive Squalomix (https://transcriptome.riken.jp/squalomix/) [40]. Amino acid and lipid transporters expressed in the intestine and the YSM were identified from transcriptome data.

### Molecular phylogenetic analysis

Molecular phylogenetic analyses were conducted with MEGA X [41]. The deduced amino acid sequences of catshark cathepsin L1 and cathepsin L2 were aligned with cathepsin homologs of other animals using MUSCLE [42]. The substitution model and parameters for inferring phylogenetic tree were chosen as previously described [13].

### Real-time quantitative PCR assay

The embryonic intestine was obtained from juveniles and embryos of stages 29, 30, 31E, 31L, 32, 33 and 34 (*N* = 5). Two layers of the YSM were sampled from stages 28, 29, 30, 31E, 31L, 32, and 33 (*N* = 5). To examine digestive capacity of the embryonic pancreas, the pancreas was dissected out from juveniles and embryos of stages 31E, 31L, 32, 33, and 34 (*N* = 5). Total RNA extraction and first-strand cDNA synthesis were conducted as previously described [13]. Quantitative PCR (qPCR) was performed using a 7900 HT Fast Real Time PCR System (Life Technologies, Gaithersburg, MD, USA) with KAPA SYBR Fast qPCR Kit (Kapa Biosystems, Wilmington, MA, USA), as previously described [13]. Primers used for real-time qPCR were designed using PrimerQuest® program (IDT, Coralville, IA, USA) (https://www.idtdna.com/SciTools.) (S1 Table).

### *In situ* hybridization

The intestine of stage 33 and YSM of stages 31E and 33 embryos were used for *in situ* hybridization. Tissues were fixed with modified Bouin’s fixative (formalin:saturated picric acid = 1:3) at 4°C overnight. For the YSM, the whole yolk sac was fixed and then trimmed to remove the vast majority of yolk. Digoxigenin (DIG)-labeled cRNA probes were synthesized from plasmids containing cDNAs encoding partial slc43a2, ApoB, CTSL1 and CTSL2. The fixed tissues were then processed for *in situ* hybridization as previously described [13].

### Transmission electron microscopy

For transmission electron microscopy observation, embryos of stages 29, 31E and 33 were examined. The inner layer of the YSM was dissected and fixed in fixative containing 2% paraformaldehyde and 2.5% glutaraldehyde in 0.05 mol l^−1^ cacodylate buffer (CB, pH 7.4) for 2h. After washing in CB for 5 min, samples were additionally fixed in the 1% osmium tetroxide for 1 h. The fixed samples were dehydrated with a series of ethanol and propylene oxide, and then embedded in Epon812. Ultrathin sections were cut with a diamond knife and mounted on grids. The sections were stained with uranyl acetate and lead citrate, and were observed using a transmission electron microscope (JEM-1400, JEOL Ltd., Japan) at 80kV.

### Statistical analysis

Data are expressed as box and whisker plots. Statistical analyses were conducted using GraphPad Prism Ver. 6 for Windows (Graph Pad software Inc., San Diego, CA, USA). The statistical significance of the difference in the expression level between each developmental stage was tested by one-way ANOVA, followed by Tukey-Kramer’s multiple comparison test. *P*-values less than 0.05 were considered statistically significant.

## Results

### Expression profiles of amino acid transporters in the intestine and the yolk sac membrane of the cloudy catshark embryo

With the application of cut-off (contig with an TPM value of > 1 in either tissues), nineteen peptide and amino acid transporters, including *slc15a1* (*pept1*) and *slc6a19* that were previously examined [13], were identified (Table 1). The genes were classified into three groups in relation to their putative intracellular localization: apical, basolateral and other localities (expressed in non-epithelial cells or localization was not determined) (Table 1) [43]. Putative apical transporters (*slc15a1* (*pept1*), *slc3a1*, *slc6a19*, *slc7a9*) and basolateral transporters (*slc3a2*, *slc7a7*, *slc7a8*) were relatively high in TPM values (> 10) in the intestine of stage 32 embryos. Meanwhile, in the inner layer of stage 31E and stage32 YSM (YSMin), most of the apical transporters were expressed at low levels (TPM values < 5), except *slc7a9* which was highly expressed. However, the basolateral and non-epithelial transporters such as *slc3a2*, *slc7a8*, *slc43a2*, and *slc7a2* showed relatively high expression (TPM values > 10).

### Developmental changes in expression levels of amino acid transporters

**Fig 2:**
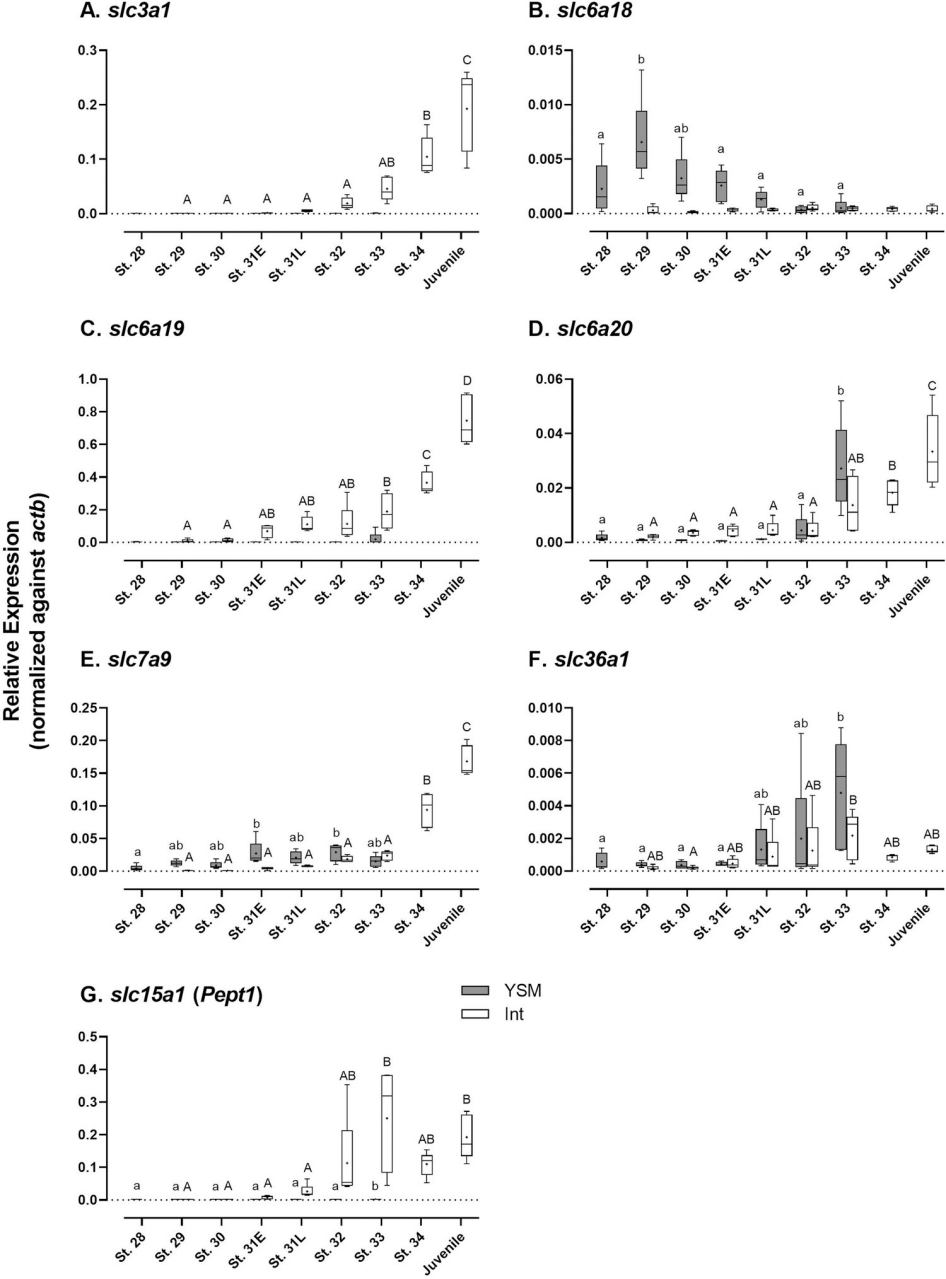
Developmental changes in gene expression of amino acid transporters of putative apical locality. (A) *slc3a1*; (B) *slc6a18*; (C) *slc6a19*; (D) *slc6a20*; (E) *slc7a9*; (F) *slc36a1*; (G) *slc15a1* (*Pept1*). Data are presented using box and whisker diagrams of *N* = 5. Uppercase and lowercase letters indicate significant developmental differences in embryonic intestine and yolk sac membrane, respectively after one way ANOVA, Tukey’s test (*P*<0.05).

**Fig 3:**
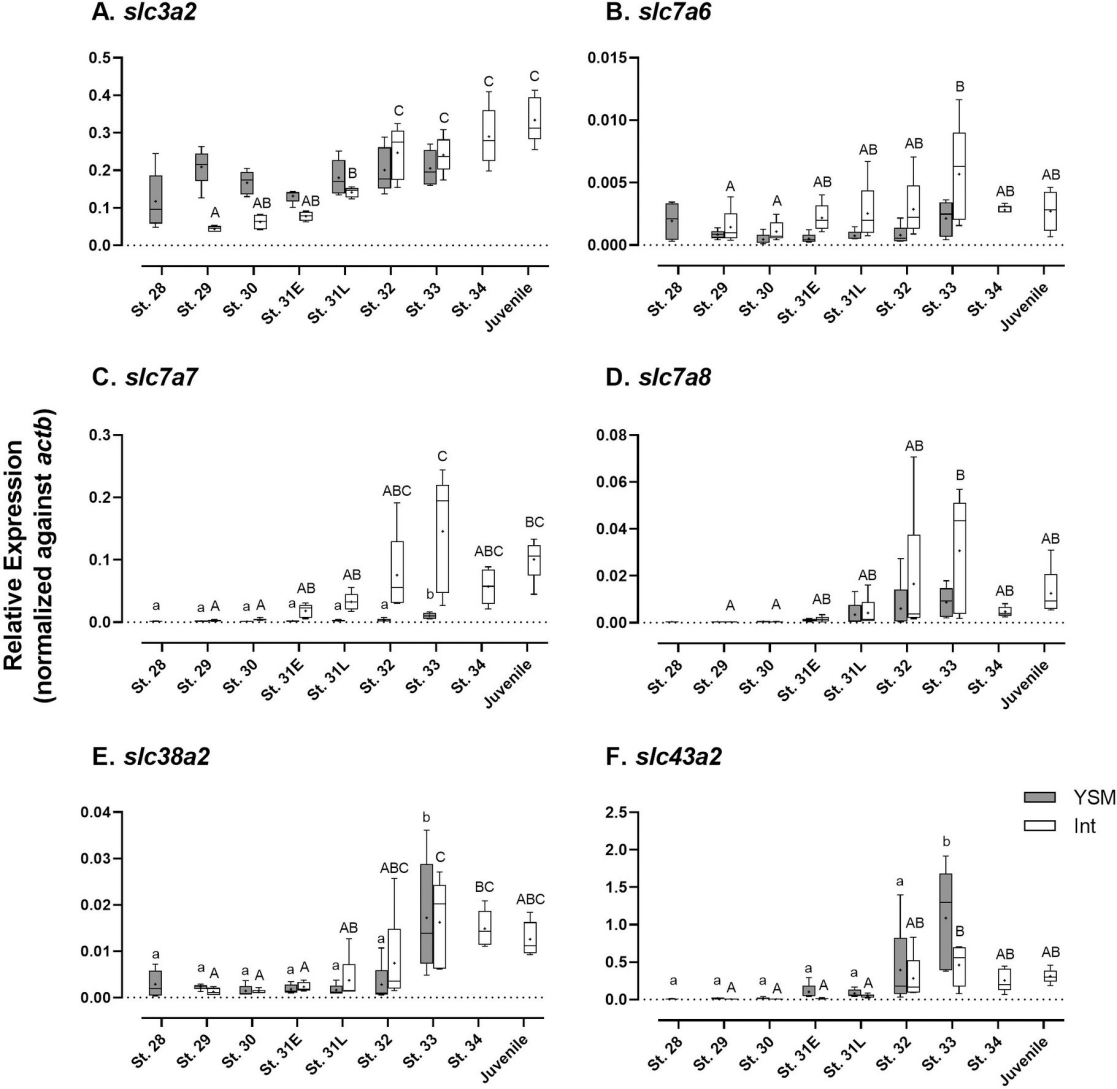
Developmental changes in gene expression of amino acid transporters of putative basolateral locality. (A) *slc3a2*; (B) *slc7a6*; (C) *slc7a7*; (D) *slc7a8*; (E) *slc38a2*; (F) *slc43a2*. Data are presented using box and whisker diagrams of *N* = 5. Uppercase and lowercase letters indicate significant developmental differences in embryonic intestine and yolk sac membrane, respectively after one way ANOVA, Tukey’s test (*P*<0.05).

The developmental changes in gene expression of amino acid transporters were examined by quantitative RT-PCR (qPCR) in the intestine (from stage 29 to hatched juvenile) and the YSM (from stages 28 to 33) (Figs 2, 3 and S1). After pre-hatching period (from stage31L), the mRNA levels of most apical amino acid transporters gradually increased in the intestine until hatching, except *slc6a18* which remained low expression throughout the development (Fig 2). In particular, the expression levels of *slc3a1*, *slc6a19*, *slc6a20* and *slc7a19* in the intestine at stage 34 and juveniles were significantly higher than those in other developmental stages. Although *slc3a1*, *slc6a19* and *pept1* remained low in expression in the YSM throughout development, other apical transporters were moderately expressed in the YSM. The mRNA levels of *slc6a20* and *slc36a1* were significantly increased in the YSM after the pre-hatching stage (Fig 2D and 2F). Unlike other apical transporters, the expression of *slc6a18* was high in the YSM before the pre-hatching stage, peaked at stage 29 and then gradually decreased after stage 32, and remained low for the rest of development (Fig 2B).

In the intestine, most of the basolateral amino acid transporters, including *slc7a7* and *slc7a8*, shared a similar expression pattern, in which the expressions were increased after pre-hatching and peaked at stage 33, and then decreased to lower levels at later stages (Fig 3B–3F). Only *slc3a2* mRNA levels were continuously increased in the intestine up to the juvenile stage (Fig 3A). In the YSM, mRNA levels of *slc38a2* and *slc43a2* markedly increased after pre-hatching (Fig 3E and 3F), whereas the *slc7a6*, *slc7a7* and *slc7a8* mRNAs remained at low levels throughout development (Fig 3B–3D). The mRNAs of *slc3a2* were highly expressed in the YSM before pre-hatching and remained at a high level until stage 33 (Fig 3A).

Consistent with the RNA-seq data, the expressions of amino acid transporters of unknown locality were at low levels in both the intestine and YSM, except for the case of *slc7a2* (S1 Fig). *slc7a2* was expressed constantly at low levels in the intestine, but the expression in the YSM continuously increased after pre-hatching (S1B Fig).

### Expression of candidate genes involved in lipid absorption

**Fig 4:**
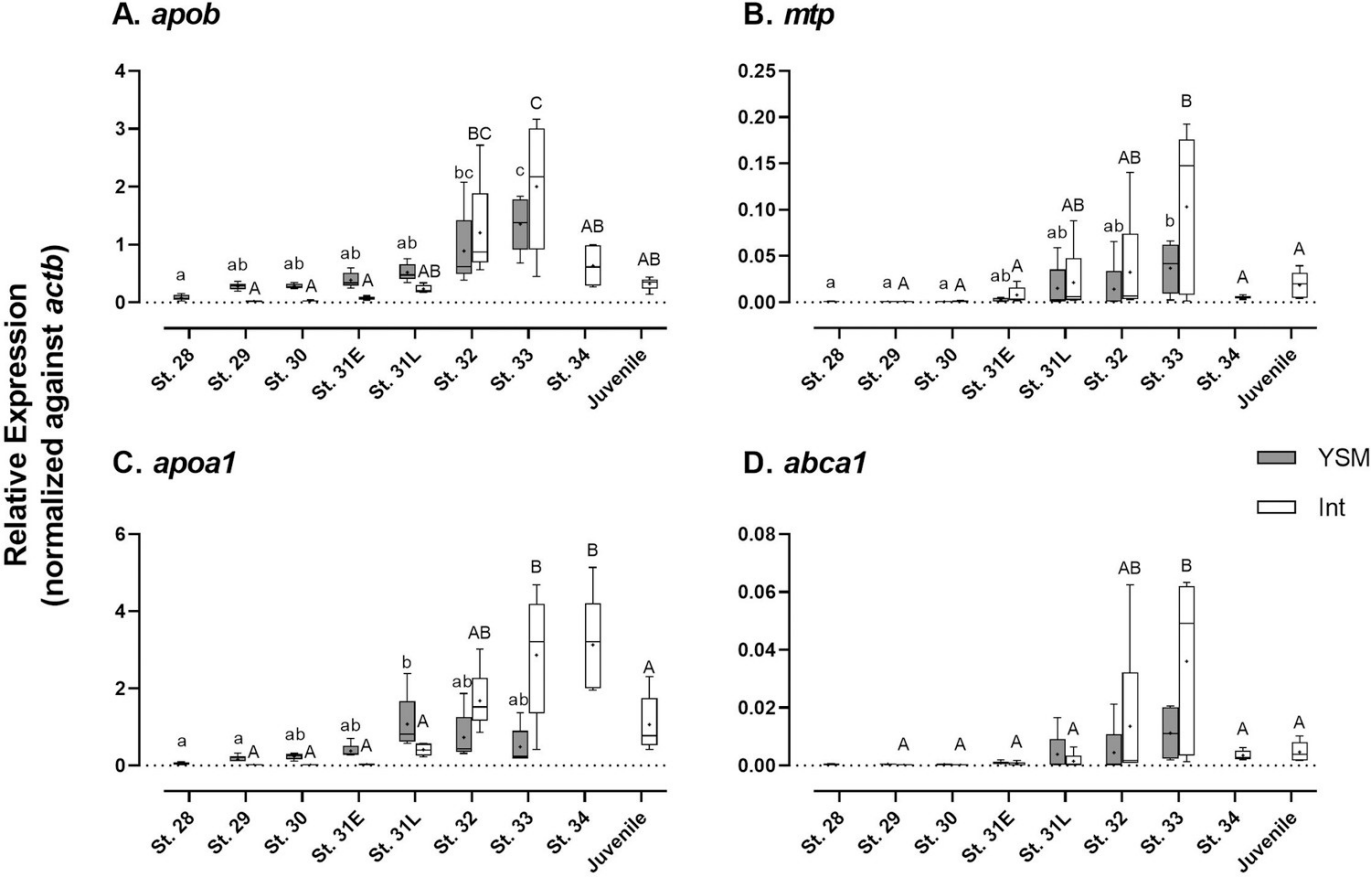
Developmental changes in gene expression involved in lipid absorption. (A) *apob*; (B) *mtp*; (C) *apoa1*; (D) *abca1*. Data are presented using box and whisker diagrams of *N* = 5. Uppercase and lowercase letters indicate significant developmental differences in embryonic intestine and yolk sac membrane, respectively after one way ANOVA, Tukey’s test (*P*<0.05).

In the intestine, all genes involved in lipid absorption were expressed in low levels in early development but were largely upregulated after pre-hatching (Fig 4). After reaching a peak level at stage 33 or 34, the expression levels then significantly decreased (Fig 4). Similar to the intestine, the lipid absorption genes in the YSM were also gradually upregulated throughout development. Meanwhile, moderate expression levels of *apob*, *apoa1* were evident in the YSM in the early developmental phase (Fig 4A, 4C and 4D). The mRNA levels of *apob*, *mtp*, and *abca1* in the YSM at stage 33 were significantly higher than those of early stages (Fig 4A, 4B and 4D), except that *apoa1* expression was peaked at stage 31L (Fig 4C).

### Gene expression of lysosomal digestive enzymes

Cloudy catshark cathepsin F1, F2, L1 and L2 were named according to the sequence similarity and the molecular phylogenetic relationships with other vertebrate cathepsin proteins (S2 Fig). A molecular phylogenetic tree showed that catshark cathepsin L1 was clustered in the clade of cathepsin L1 of other vertebrates. Catshark cathepsin L2 was not clustered with any clades and was branched outside of the clade of cathepsin L1, cathepsin K, and cathepsin S. Catshark cathepsin F1 and cathepsin F2 were clustered in the clade of cathepsin F.

**Fig 5:**
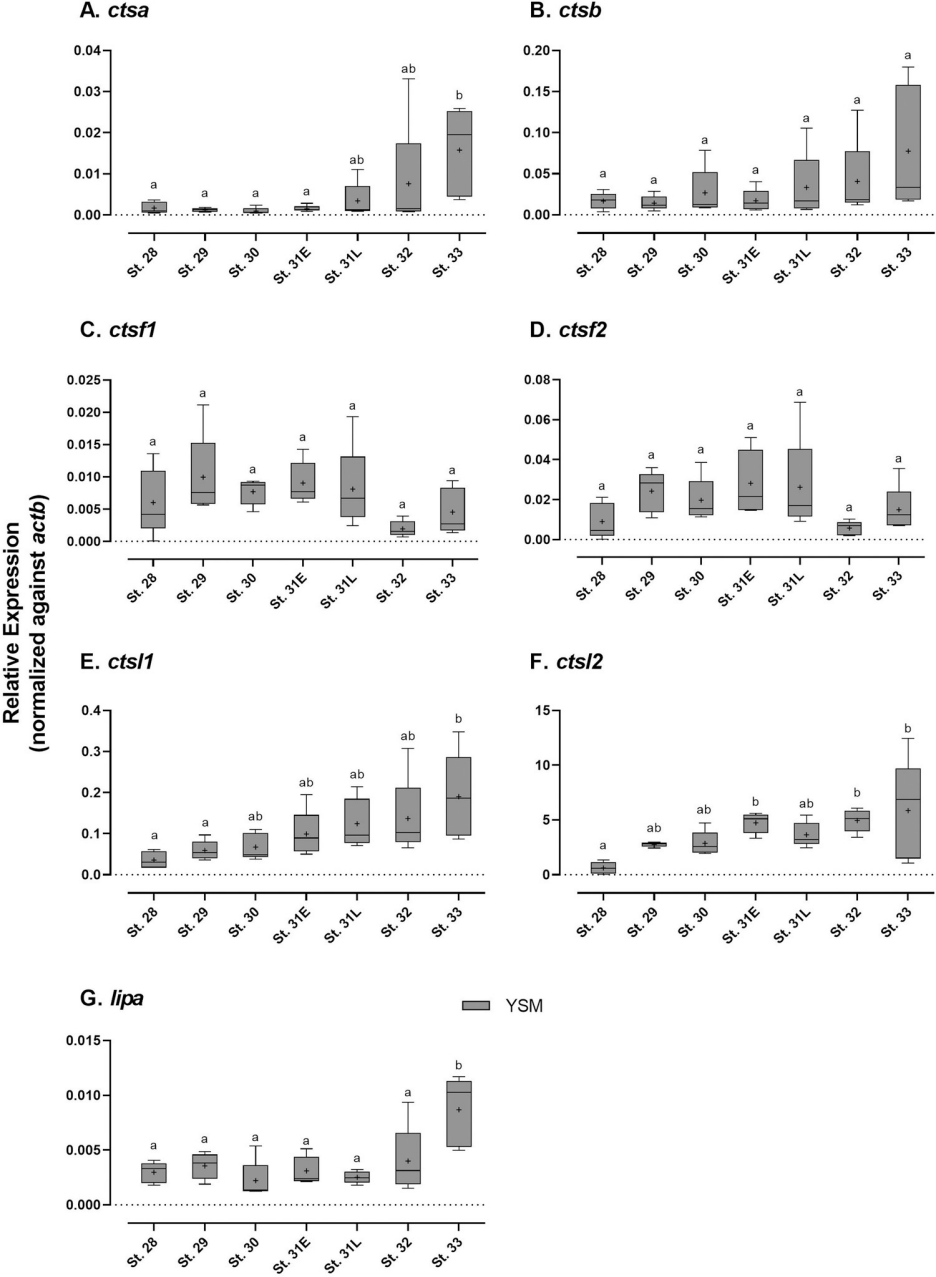
Developmental changes in gene expression of lysosomal digestive enzymes. (A) *ctsa*; (B) *ctsb*; (C) *ctsf1*; (D) *ctsf2*; (E) *ctsl1*; (F) *ctsl2*; (G) *lipa*. Data are presented using box and whisker diagrams of *N* = 5. Uppercase and lowercase letters indicate significant developmental differences in embryonic intestine and yolk sac membrane, respectively after one way ANOVA, Tukey’s test (*P*<0.05).

During development, *ctsl1*, *ctsl2*, and *ctsb* mRNAs were expressed at considerably high level (Fig 5B, 5E and 5F). The expression of *ctsl2* was 10 to 1000 times higher than those of other cathepsin mRNAs, consistent with transcriptome data (Table 2 and Fig 5F). Although the mRNA levels of *ctsf1* and *ctsf2* fluctuated, expressions of other cathepsins and *lipa* continuously increased in the YSM throughout development (Fig 5).

### Localization of *slc43a2* and *apob* mRNAs in yolk sac membrane and intestine

**Fig 6:**
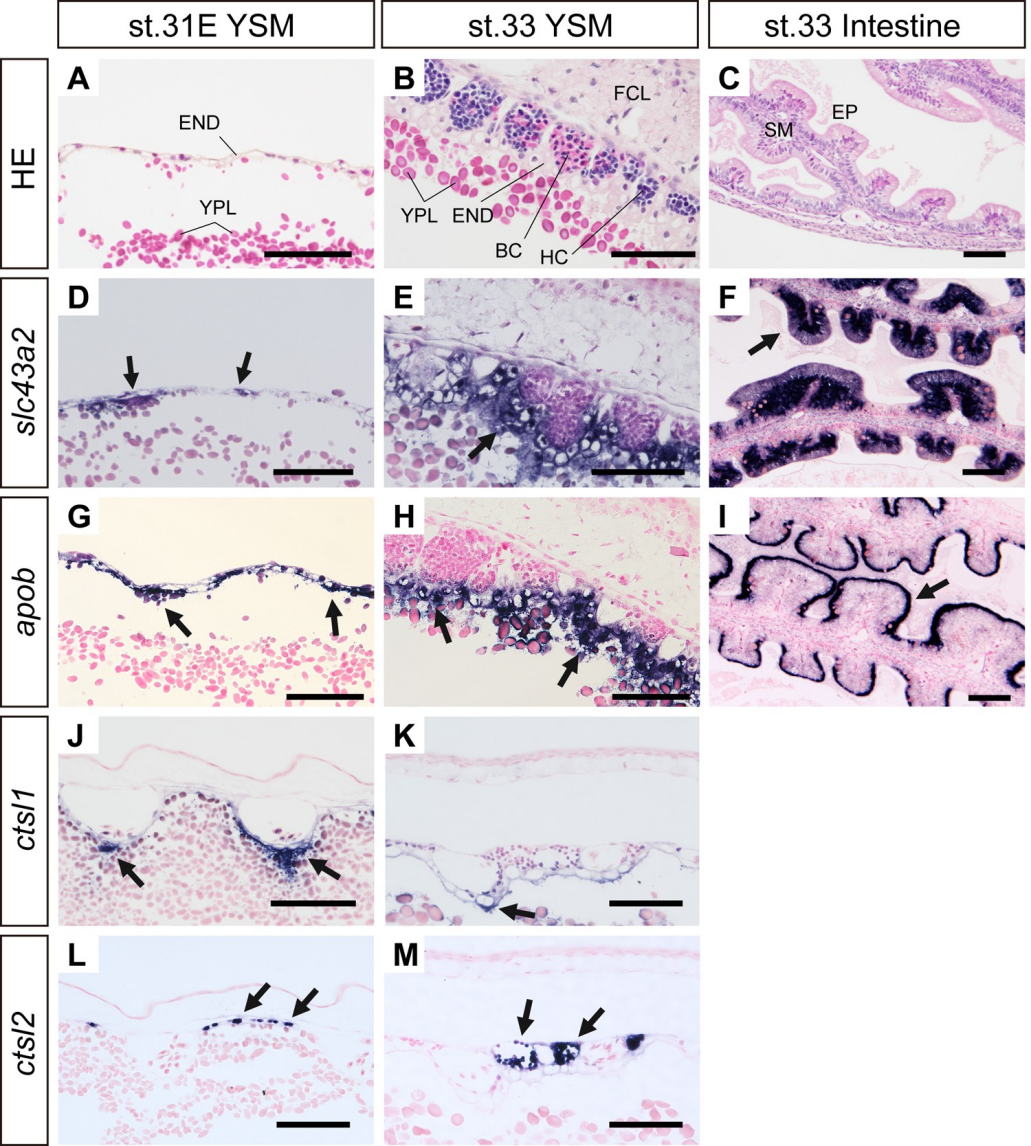
Localization of the mRNAs involved in lipid absorption and lysosomal digestive enzyme in the yolk sac membrane. Morphological observations of the YSM shown by hematoxylin and eosin staining (A-C). *In situ* hybridization staining of *slc43a2* (D-F), *apob* (G-I), *ctsl1* (J, K), and *ctsl2* (L, M). Arrows indicate the positive signals. END, endoderm; EP, epithelial cells; FCL, fibrous connective layer; HC, hematopoietic cell; SM, submucosa; BC, blood cells. Bars, 100μm.

Based on the results of qPCR analyses, *in situ* hybridization was performed to examine the localization of abundantly expressed *slc43a2* and *apob* mRNAs. In the YSM, both *slc43a2* and *apob* mRNA signals were detected in the endodermal cells that are in contact with yolk platelets (Fig 6D, 6E, 6G and 6H), and no signal was observed in hematopoietic cells or fibrous connective layer (Fig 6E and 6H). In the intestine of stage 33 embryos, both *slc43a2* and *apob* mRNA signals were detected in the epithelial cells (Fig 6F and 6I), and signals of *apob* were restricted just below the apical surface of epithelial cells (Fig 6I).

### Localization of *ctsl1* and *ctsl2* mRNAs in the yolk sac membrane

Among the cathepsin mRNAs, localization of *ctsl1* and *ctsl2* mRNAs were investigated (Fig 6J–6M). The *ctsl1* mRNA signals were detected in the endodermal cells and yolk syncytial layer of the YSM (Fig 6J and 6K). Meanwhile, *ctsl2* mRNA signals were observed in the hematopoietic cells (Fig 6L and 6M), and no signal was observed in endodermal cells or blood cells.

### Endocytosis of yolk droplets into the endodermal cells

**Fig 7:**
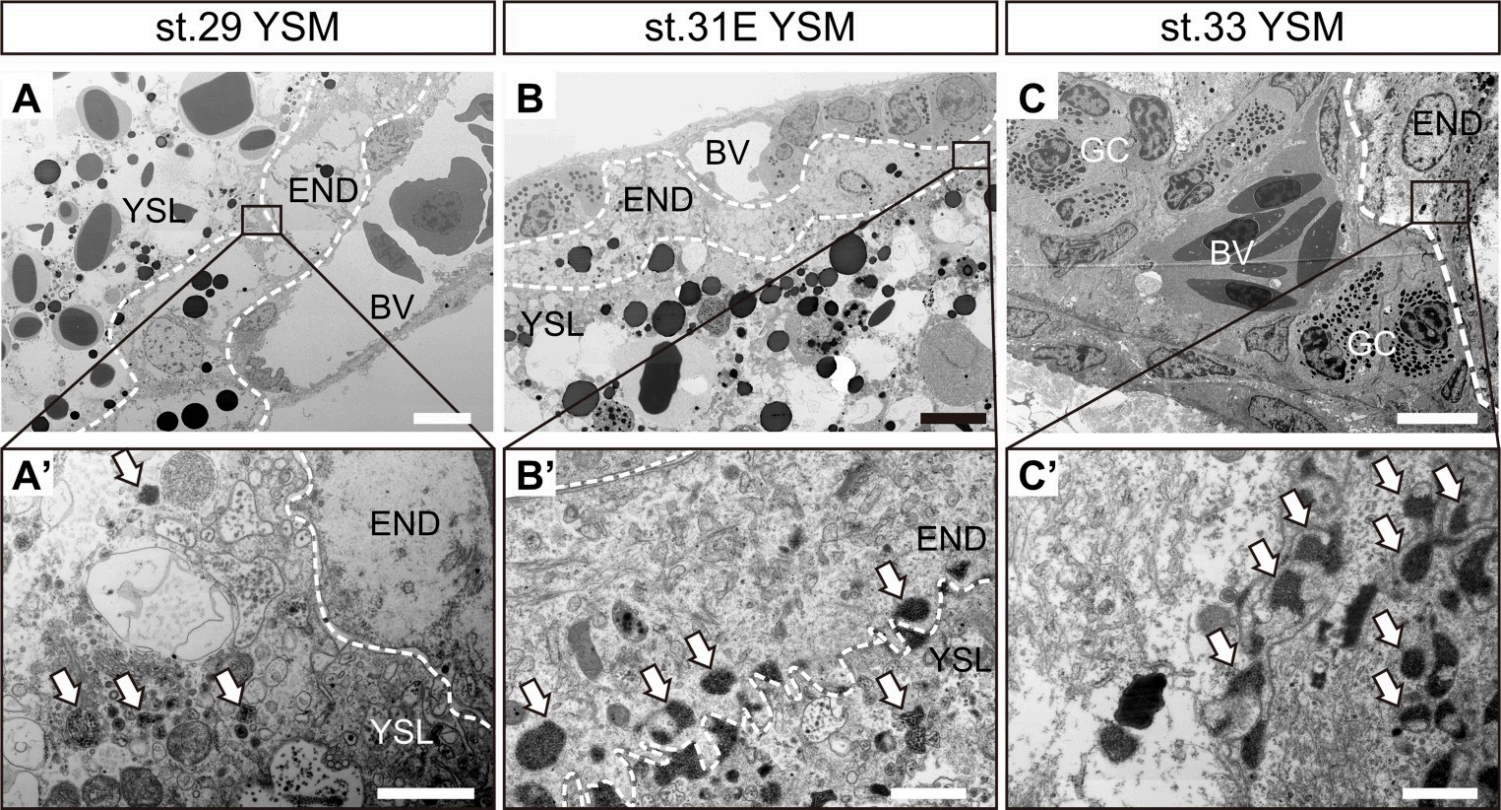
Electron microscopic ultrastructural observations on the developing yolk sac membrane. (A-C) Ultrastructure of the boundary between yolk contents and epithelial cells of the YSM in different developmental stages. (A’-C’) Small boxes in panels A-C are magnified to show the fine structures. Arrows indicate membrane packets containing yolk granules. BV, blood vessels; END, endodermal cells; GC, granular cells; YSL, yolk syncytial layer. Bars, 10μm (A-C), 1μm (A’-C’).

To investigate endocytosis of yolk granules, TEM observation was performed (Fig 7). Small yolk granules enveloped in a membrane structure were observed in the YSL near the boundary with the endodermal cell at stage 29 (Fig 7A and 7A’, arrows). Both the number of membrane packets containing yolk droplets and the density of yolk droplets inside the packets increased at stage 31E (Fig 7B and 7B’, arrows). Membrane packets containing yolk granules were highly abundant at stage 33 (Fig 7C and 7C’, arrows).

### Expression levels of mRNAs encoding pancreatic digestive enzymes

In order to investigate the digestive capacity of the embryonic pancreas, mRNA levels of digestive enzymes including trypsin, bile salt-activated lipase (*bsdl*), and phospholipase A2 (*pla2g1b*) were quantified (S3 Fig). The *trypsin* and *pla2g1b* mRNA levels were low in stages 31E and 31L, and then increased after stage 32 until hatched juvenile stage (S3A and S3B Fig). The *bsdl* mRNA expression was low until stage 34, but was significantly increased in hatched juveniles (S3C Fig).

### Developmental changes in the wet weight of embryo and yolk sac

Wet weight of the embryo and the yolk sac containing the yolk were measured (S4 Fig). The respective weight gain and loss of the embryo and yolk sac we measured were very small until stage 31L, and they became statistically significant only after pre-hatching.

## Discussion

The present study examined the expression profiles of the genes involved in nutrient metabolism in both the embryonic intestine and YSM during the development of the oviparous cloudy catshark. Since we previously found that the embryonic intestine of the catshark becomes functional at the pre-hatching stage and starts absorbing nutrients from the yolk in the subsequent stages [13], we hypothesized that the site for nutrient absorption switches from the YSM to the intestine around pre-hatching. However, contrary to our hypothesis, the YSM is likely contributing to embryonic nutrition along with the intestine, particularly after pre-hatching.

### Nutrient absorption by the YSM

In addition to the embryonic intestine, the yolk-sac lining is also a major site for nutrient absorption in oviparous vertebrates [44,45]. Indeed, in several shark species, the yolk platelets are first degraded in the yolk syncytial layer, and the resulting yolk granules are incorporated into endodermal cells by endocytosis [7,15–17]. The membrane packets containing yolk droplets were also observed in the endodermal cells of YSM from stage 29 to 33 in cloudy catshark, suggesting that the endocytosis from yolk syncytial layer to the endodermal cell occurs at least after stage 29. In aplacental viviparous cartilaginous fishes, the YSM contributes to nutrient absorption during the early stages of development. Following this, the embryonic intestine takes over the role as the major organ absorbing nutrients late in development [14,15]. However, we found that the density of yolk granules inside the membrane packet increased after pre-hatching, and that the expression of many amino acid transporters and lipid absorption molecules in the YSM continuously increased after pre-hatching in the cloudy catshark. Coinciding with the present findings, an ultrastructural study of the YSM of the oviparous lesser spotted dogfish (*S*. *canicula*) suggested that the absorption of yolk nutrients was enhanced after pre-hatching [7]. Therefore, these results suggest that nutrient absorption activities are low in the YSM during early development, and gradually increased in late development of oviparous cartilaginous fishes. The differences in the timing of nutrient absorption by the YSM between Scyliorhinidae and *S*. *acanthias* could be due to the different nutrient supplying systems. Since the *S*. *acanthias* embryos are nourished by additional histotroph [2,46–48], yolk nutrients are likely essential only in the early developmental phase as embryonic nutrition subsequently transits to histotrophy in viviparous species.

Yolk lipoproteins are likely digested by cathepsin proteases in the endodermal cells of the YSM in catshark. Four (*ctsa*, *ctsb*, *ctsl1*, *ctsl2*) out of six cathepsins examined have increased expression throughout development, particularly after pre-hatching, and the mRNA signal of *ctsl1* was abundantly detected at the endodermal epithelia of the YSM. These findings suggest a digestive role of cathepsins on yolk granules in the YSM. The involvement of CTSA, CTSB, and CTSL in yolk protein digestion is also found in chicken and several teleost fishes [19,49–52]. Meanwhile, catshark *ctsl2* mRNA signal was not detected in the endodermal layer but in the hematopoietic cells of the YSM, which is reasonable as several cathepsins are known to play critical roles in hematopoietic cell differentiation in mammals [53,54].

Similar to the results of basolateral amino acid transporters, high expression levels of *ctsa*, *ctsb*, and *ctsl1* were observed in the YSM in late developmental stages. As in mammals where the *slc43a2* is critically important for the intrauterine growth of embryos [55,56], catshark *slc43a2* was also found to be the most abundantly expressed basolateral transporters examined in this study, and its expression was markedly increased after pre-hatching. Furthermore, all examined genes involved in the lipid absorption were also upregulated in the YSM after pre-hatching. Therefore, these results infer that the amino acids and lipid derived from the digested yolk granules in the epithelial cells are transported through the basolateral membrane of YSM into the blood circulation.

### Amino acid and lipid metabolism in the embryonic intestine

Unlike the YSM, the embryonic intestine expressed both apical and basolateral amino acid transporters and most transporters tended to be upregulated concomitantly after pre-hatching. These results suggest that free amino acids in the yolk are likely absorbed transcellularly in the intestine. Similar expression levels and patterns of the orthologues of the examined genes were reported in studies of mammalian intestinal epithelial cells [43,57]. We also found that the gene expression of pancreatic digestive enzymes such as trypsin and phospholipase A2 were increased in the embryonic pancreas after pre-hatching. In *S*. *acanthia*s, zymogen granules were observed in the pancreas of 65 mm embryos (presumably correspond to stage 31L of cloudy catshark) when the yolk begins to flow from the EYS into the intestine through the yolk stalk [14]. Therefore, together with our previous findings [13], the embryonic intestine may actively digest proteins and phospholipids of the yolk and absorb the nutrients after pre-hatching.

Interestingly, pancreatic expression of cholesterol esterase (*bsdl*) was maintained at the low level during embryonic development, and it markedly increased in hatched juveniles that were fed with squid. As squid is a diet of high cholesterol, the increased *bsdl* expression could be a physiological response to digest cholesterol. The lipid content of the egg yolk of *S*. *canicula* is composed of 30–60% phospholipid, only 5% cholesterol [58]. As the cholesterol is low in the yolk, it is reasonable that the expression pattern of *bsdl* is different from those of the trypsin and phospholipase A2.

It is also noteworthy that the expressions of many genes responsible for nutrient absorption were decreased in the intestine at stage 34 just before hatching. In particular, *apob* and *mtp*, which are essential for chylomicron assembly during lipid absorption, were [37,59] significantly downregulated in the embryonic intestine around hatching. Meanwhile, four out of seven apical amino acid transporters showed continuously increasing expression until the juvenile stage. These findings suggest that transfer of amino acids and of lipids into blood circulation is slowed down by the downregulation of basolateral transporters just before the hatching, while apical transporters remains functional. This sequence of events likely leads to the accumulation of the nutrients in the intestinal epithelial cells. A similar observation has been made in the embryonic intestine of freshwater stingray where yolk-like substances were accumulated within the epithelial cells in late developmental stages [60]. The retention of nutrients in the intestinal epithelium before hatching or parturition could be advantageous for survival until the juveniles start to feed by themselves.

### Contribution of the yolk nutrients to growth and development

After pre-hatching, the egg yolk inside the EYS begins to flow into the embryonic intestine and there was a significant increase in the rate of body weight in catshark embryos. A similar pattern in the yolk consumption during the embryonic period (slow in early period and fast in later period) is also observed in Japanese quail (*Coturnix japonica*) [61] and European plaice (*Pleuronectes platessa*) [62]. In other cartilaginous fishes, additional maternal nutrient supplies to the embryos are commonly found in viviparous species, and these nutrients are known to contribute the rapid embryonic growth in late developmental stages [2,48,63]. For example, embryos of great white shark (*Carcharodon carcharias*) ingest the maternal histotroph and digest nutrients by the gastrointestinal tract [2]. Together, these findings suggest that the growth rate of many vertebrates increases in late development after the embryonic alimentary canal acquires an ability to digest and absorb the maternally-derived nutrient sources in both oviparous and viviparous species.

In summary, the protein/lipid digestion and absorption are likely activated after pre-hatching in both the intestine and the YSM of oviparous cloudy catshark. The nutrient absorption mechanisms between these tissues are different where the YSM is likely absorbing via both endocytic and transcellular pathways, while the embryonic intestine performs transcellular membrane transport. The gene upregulations involved in nutrient absorption in both intestine and YSM after pre-hatching period strongly indicate that the YSM is equally active in the late developmental phase. Both tissues utilize the yolk contents in parallel to facilitate a rapid embryonic growth of oviparous cloudy catshark.

## Supporting information

S1 Fig Developmental changes in gene expression of amino acid transporters of unknown (other) locality. (A) *slc1a6*; (B) *slc7a2*; (C) *slc7a3*; (D) *slc36a4*; (E) *slc38a5*; (F) *slc38a7*. Data are presented using box and whisker diagrams of *N* = 5. Different uppercase and lowercase letters indicate a significant developmental difference in embryonic intestine and yolk sac membrane, respectively (*P*<0.05).
(TIF)

S2 Fig Molecular phylogenetic tree of vertebrate cathepsin genes. Bootstrap probabilities are shown next to the branches. The accession numbers of the genes used in the analysis are listed in S2 Table. The cloudy catshark sequences are highlighted by the red boxes.
(TIF)

S3 Fig Developmental changes in pancreatic enzyme gene expression. (A) *trypsin*; (B) *pla2g1b*; (C) *bsdl*. The mRNA levels are shown as the relative values to the mRNA levels of β-actin (*actb*). Data are presented using box and whisker diagrams of *N* = 5. Different lowercase letters indicate a significant difference (*P*<0.05).
(TIF)

S4 Fig Developmental changes in the wet weight of embryo and external yolk sac. Data are presented using box and whisker diagrams of *N* = 5. Different uppercase and lowercase letters indicate a significant developmental difference in embryo and external yolk sac, respectively (*P*<0.05).
(TIF)

S1 Table Primer sets used in this study. (PPTX)

S2 Table Accession numbers of the genes used in the phylogenetic analysis. (PPTX)
