# Supplementary figures and images for: Molecular mechanism of nutrient uptake in developing embryos of oviparous cloudy catshark (Scyliorhinus torazame)
Source: PLoS One. 2022 Mar 15;17(3):e0265428. doi: 10.1371/journal.pone.0265428 (PMC8923501; doi:10.1371/journal.pone.0265428)

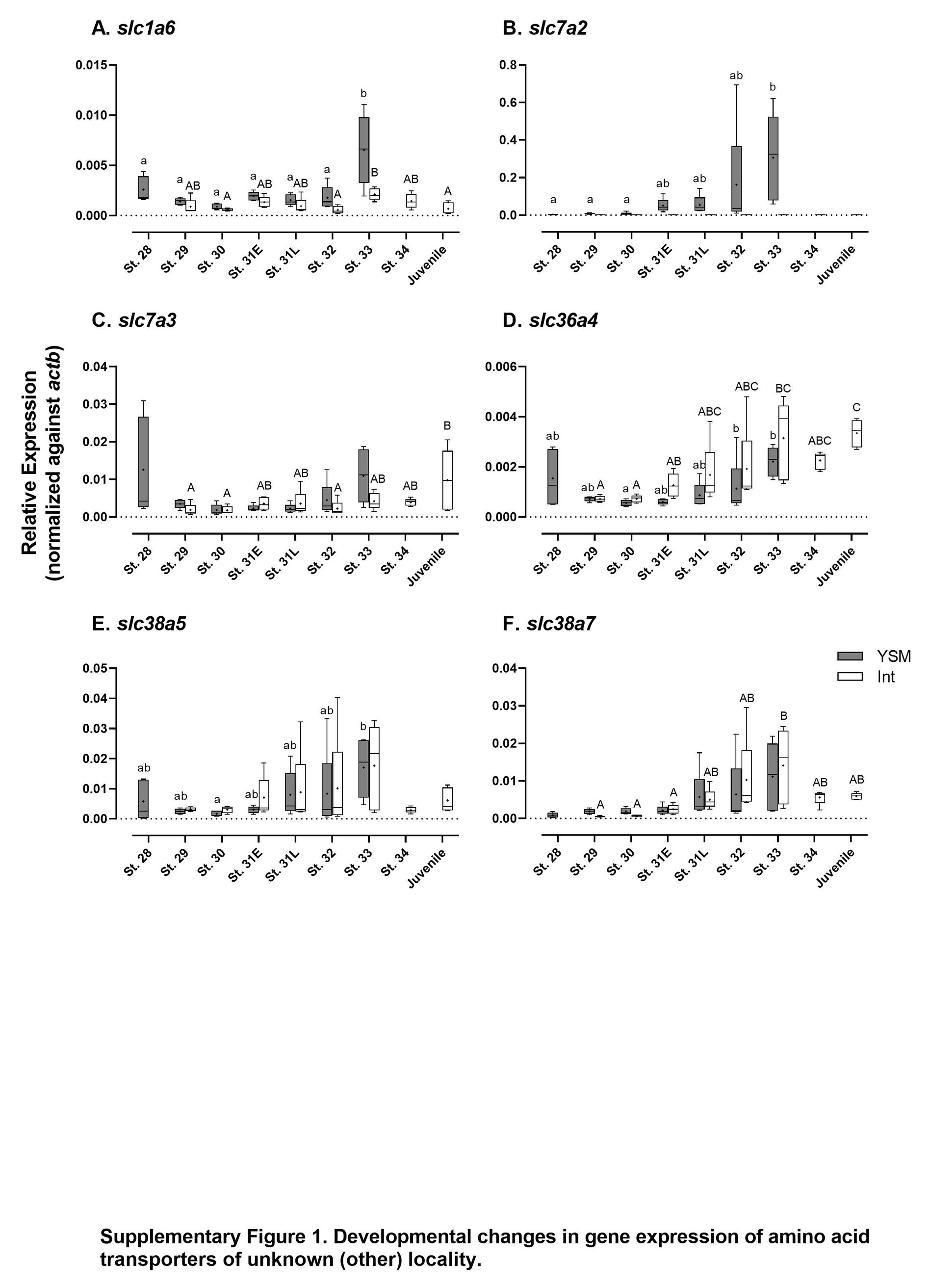

Supplement: S1 Fig — (A) slc1a6; (B) slc7a2; (C) slc7a3; (D) slc36a4; (E) slc38a5; (F) slc38a7. Data are presented using box and whisker diagrams of N = 5. Different uppercase and lowercase letters indicate a significant developmental difference in embryonic intestine and yolk sac membrane, respectively (P<0.05). (TIF) [file pone.0265428.s001.tif]

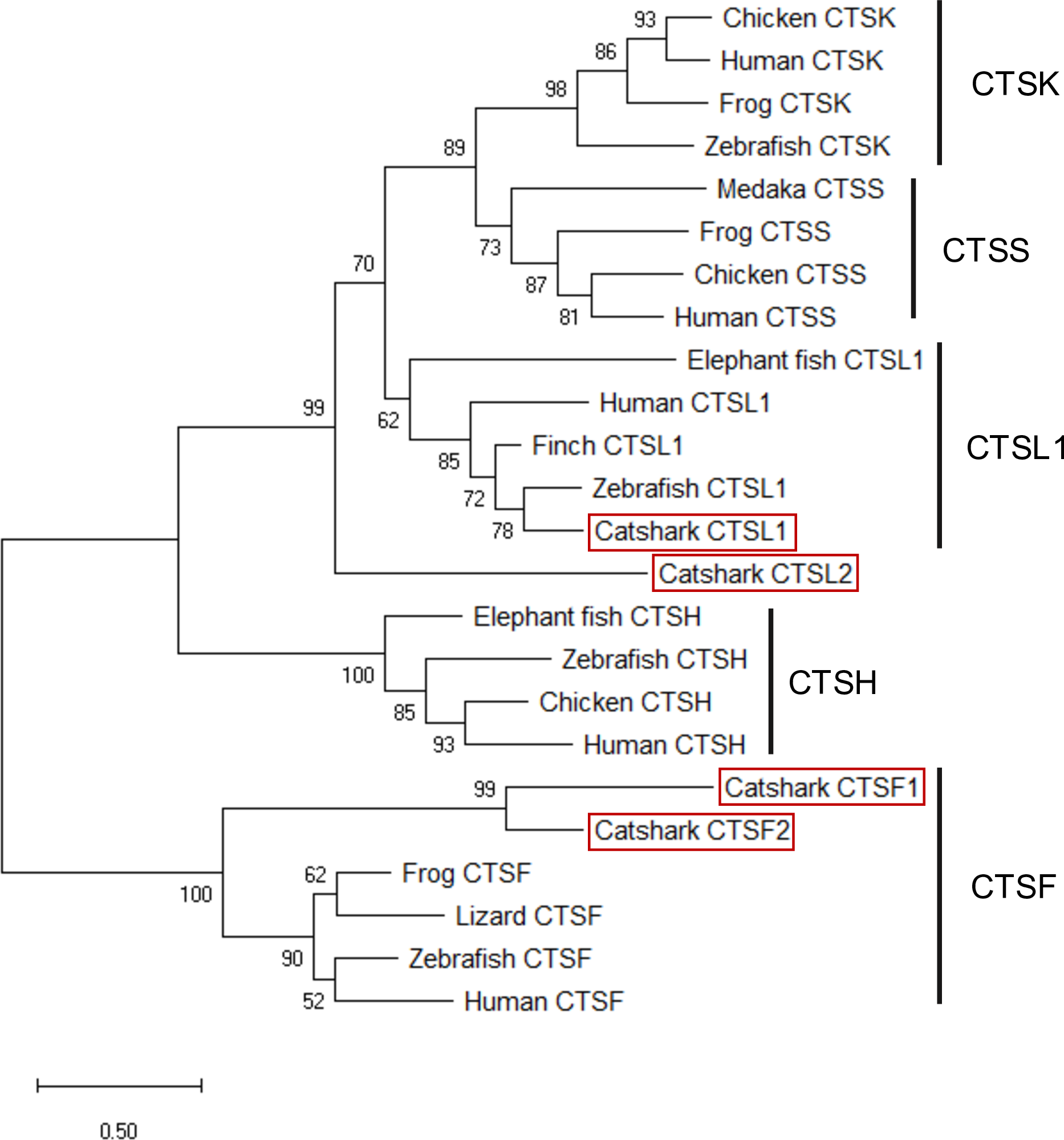

Supplement: S2 Fig — Bootstrap probabilities are shown next to the branches. The accession numbers of the genes used in the analysis are listed in S2 Table. The cloudy catshark sequences are highlighted by the red boxes. (TIF) [file pone.0265428.s002.tif]

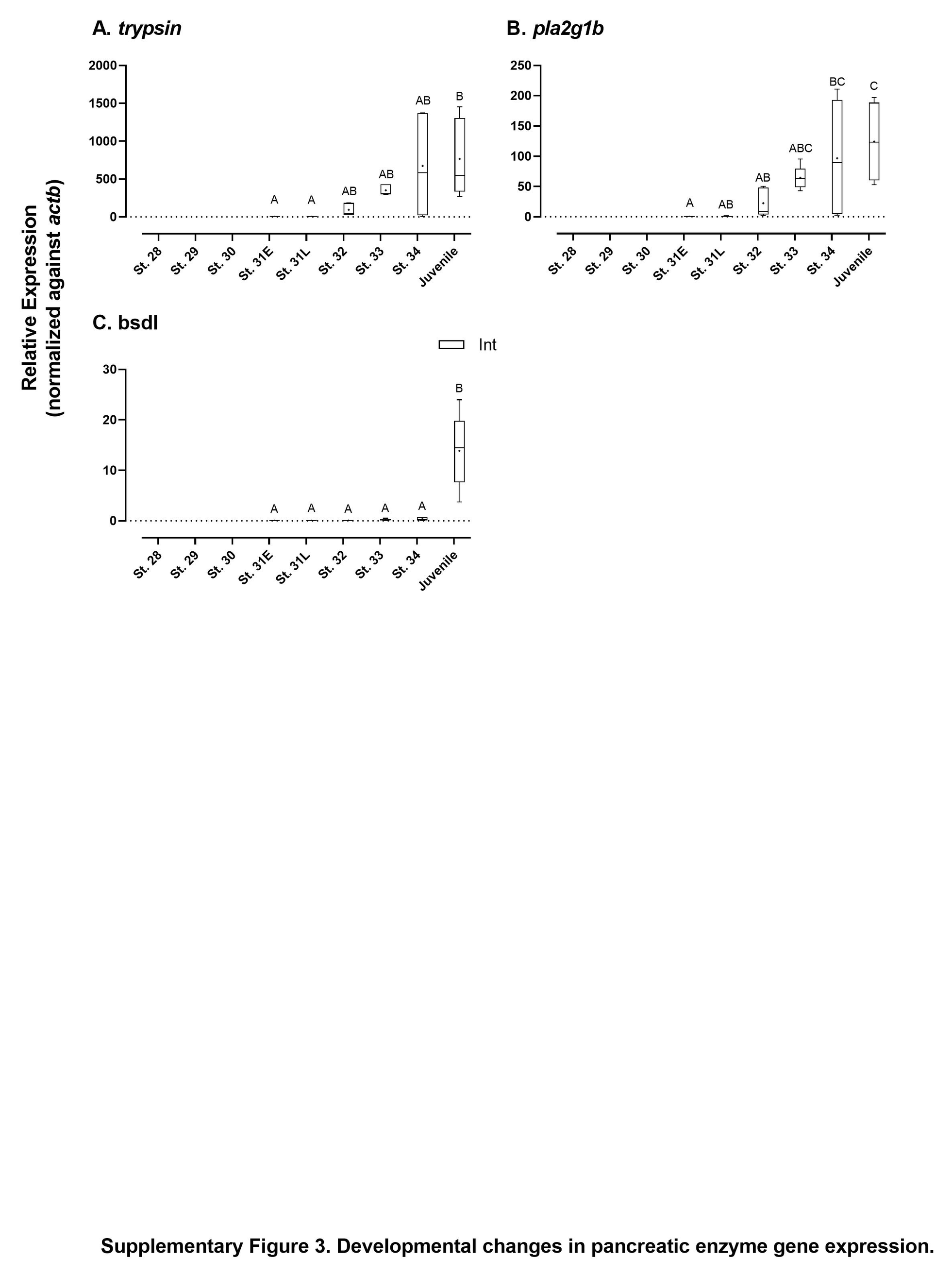

Supplement: S3 Fig — (A) trypsin; (B) pla2g1b; (C) bsdl. The mRNA levels are shown as the relative values to the mRNA levels of β-actin (actb). Data are presented using box and whisker diagrams of N = 5. Different lowercase letters indicate a significant difference (P<0.05). (TIF) [file pone.0265428.s003.tif]

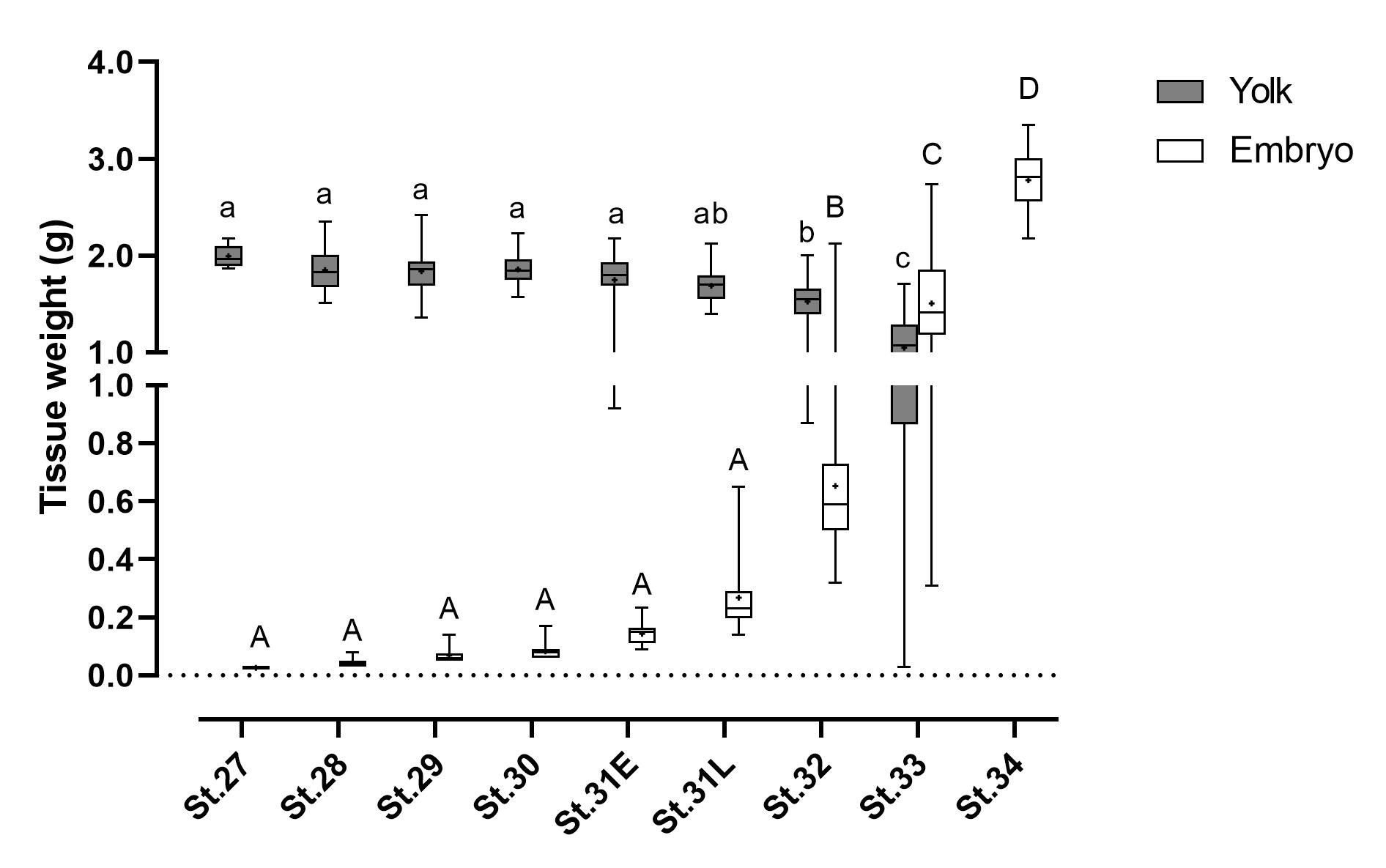

Supplement: S4 Fig — Data are presented using box and whisker diagrams of N = 5. Different uppercase and lowercase letters indicate a significant developmental difference in embryo and external yolk sac, respectively (P<0.05). (TIF) [file pone.0265428.s004.tif]
