# Supplementary material for: Molecular mechanism of nutrient uptake in developing embryos of oviparous cloudy catshark (Scyliorhinus torazame)
Source: PLoS One. 2022 Mar 15;17(3):e0265428. doi: 10.1371/journal.pone.0265428 (PMC8923501; doi:10.1371/journal.pone.0265428)
Supplement: S1 Table — (PPTX) [file pone.0265428.s005.pptx]

## Slide 1
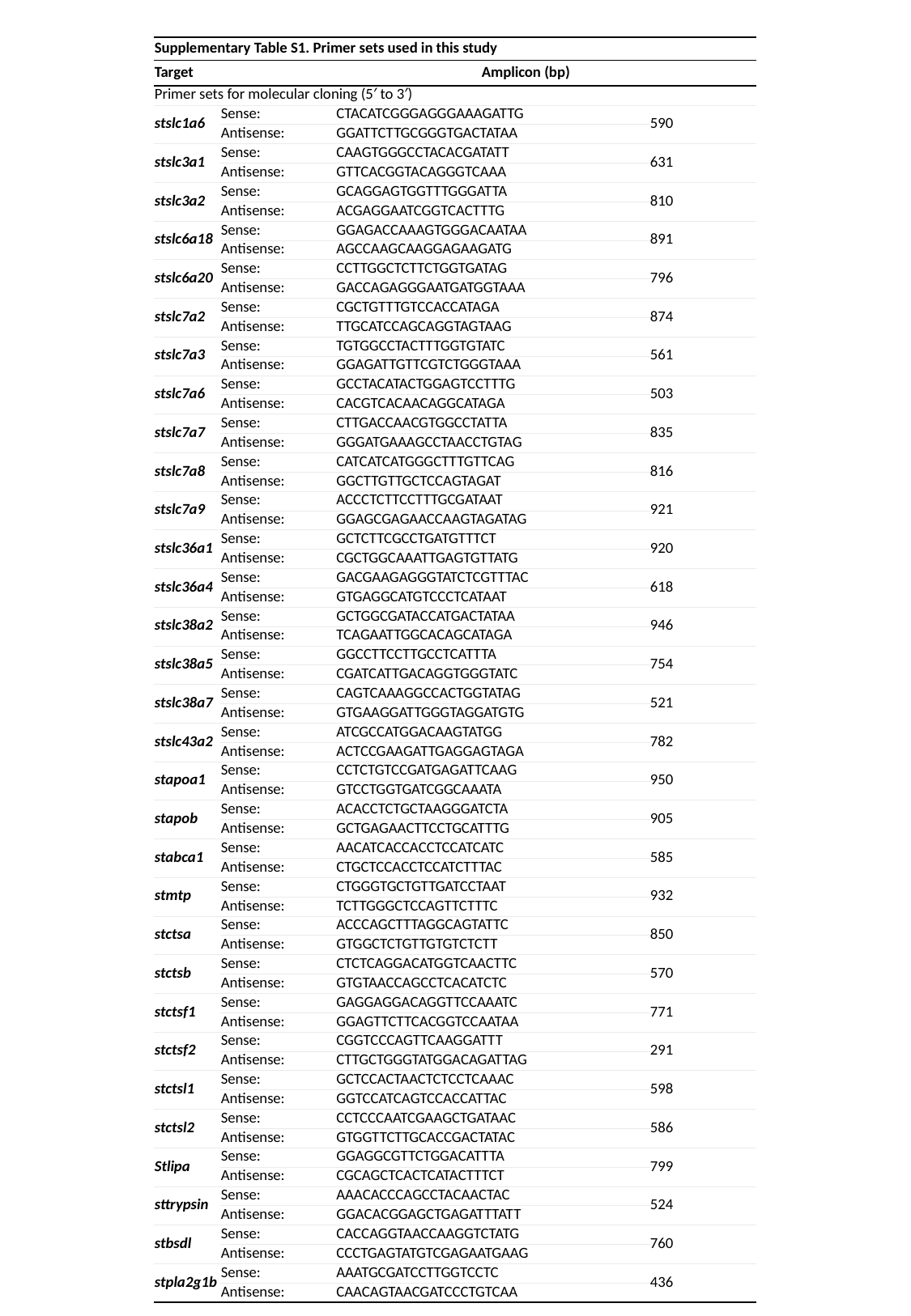

| Supplementary Table S1. Primer sets used in this study | | | | | |
| --- | --- | --- | --- | --- | --- |
| Target | | | | Amplicon (bp) | |
| Primer sets for molecular cloning (5′ to 3′) | | | | | |
| stslc1a6 | Sense: | | CTACATCGGGAGGGAAAGATTG | | 590 |
| | Antisense: | | GGATTCTTGCGGGTGACTATAA | | |
| stslc3a1 | Sense: | | CAAGTGGGCCTACACGATATT | | 631 |
| | Antisense: | | GTTCACGGTACAGGGTCAAA | | |
| stslc3a2 | Sense: | | GCAGGAGTGGTTTGGGATTA | | 810 |
| | Antisense: | | ACGAGGAATCGGTCACTTTG | | |
| stslc6a18 | Sense: | | GGAGACCAAAGTGGGACAATAA | | 891 |
| | Antisense: | | AGCCAAGCAAGGAGAAGATG | | |
| stslc6a20 | Sense: | | CCTTGGCTCTTCTGGTGATAG | | 796 |
| | Antisense: | | GACCAGAGGGAATGATGGTAAA | | |
| stslc7a2 | Sense: | | CGCTGTTTGTCCACCATAGA | | 874 |
| | Antisense: | | TTGCATCCAGCAGGTAGTAAG | | |
| stslc7a3 | Sense: | | TGTGGCCTACTTTGGTGTATC | | 561 |
| | Antisense: | | GGAGATTGTTCGTCTGGGTAAA | | |
| stslc7a6 | Sense: | | GCCTACATACTGGAGTCCTTTG | | 503 |
| | Antisense: | | CACGTCACAACAGGCATAGA | | |
| stslc7a7 | Sense: | | CTTGACCAACGTGGCCTATTA | | 835 |
| | Antisense: | | GGGATGAAAGCCTAACCTGTAG | | |
| stslc7a8 | Sense: | | CATCATCATGGGCTTTGTTCAG | | 816 |
| | Antisense: | | GGCTTGTTGCTCCAGTAGAT | | |
| stslc7a9 | Sense: | | ACCCTCTTCCTTTGCGATAAT | | 921 |
| | Antisense: | | GGAGCGAGAACCAAGTAGATAG | | |
| stslc36a1 | Sense: | | GCTCTTCGCCTGATGTTTCT | | 920 |
| | Antisense: | | CGCTGGCAAATTGAGTGTTATG | | |
| stslc36a4 | Sense: | | GACGAAGAGGGTATCTCGTTTAC | | 618 |
| | Antisense: | | GTGAGGCATGTCCCTCATAAT | | |
| stslc38a2 | Sense: | | GCTGGCGATACCATGACTATAA | | 946 |
| | Antisense: | | TCAGAATTGGCACAGCATAGA | | |
| stslc38a5 | Sense: | | GGCCTTCCTTGCCTCATTTA | | 754 |
| | Antisense: | | CGATCATTGACAGGTGGGTATC | | |
| stslc38a7 | Sense: | | CAGTCAAAGGCCACTGGTATAG | | 521 |
| | Antisense: | | GTGAAGGATTGGGTAGGATGTG | | |
| stslc43a2 | Sense: | | ATCGCCATGGACAAGTATGG | | 782 |
| | Antisense: | | ACTCCGAAGATTGAGGAGTAGA | | |
| stapoa1 | Sense: | | CCTCTGTCCGATGAGATTCAAG | | 950 |
| | Antisense: | | GTCCTGGTGATCGGCAAATA | | |
| stapob | Sense: | | ACACCTCTGCTAAGGGATCTA | | 905 |
| | Antisense: | | GCTGAGAACTTCCTGCATTTG | | |
| stabca1 | Sense: | | AACATCACCACCTCCATCATC | | 585 |
| | Antisense: | | CTGCTCCACCTCCATCTTTAC | | |
| stmtp | Sense: | | CTGGGTGCTGTTGATCCTAAT | | 932 |
| | Antisense: | | TCTTGGGCTCCAGTTCTTTC | | |
| stctsa | Sense: | | ACCCAGCTTTAGGCAGTATTC | | 850 |
| | Antisense: | | GTGGCTCTGTTGTGTCTCTT | | |
| stctsb | Sense: | | CTCTCAGGACATGGTCAACTTC | | 570 |
| | Antisense: | | GTGTAACCAGCCTCACATCTC | | |
| stctsf1 | Sense: | | GAGGAGGACAGGTTCCAAATC | | 771 |
| | Antisense: | | GGAGTTCTTCACGGTCCAATAA | | |
| stctsf2 | Sense: | | CGGTCCCAGTTCAAGGATTT | | 291 |
| | Antisense: | | CTTGCTGGGTATGGACAGATTAG | | |
| stctsl1 | Sense: | | GCTCCACTAACTCTCCTCAAAC | | 598 |
| | Antisense: | | GGTCCATCAGTCCACCATTAC | | |
| stctsl2 | Sense: | | CCTCCCAATCGAAGCTGATAAC | | 586 |
| | Antisense: | | GTGGTTCTTGCACCGACTATAC | | |
| Stlipa | Sense: | | GGAGGCGTTCTGGACATTTA | | 799 |
| | Antisense: | | CGCAGCTCACTCATACTTTCT | | |
| sttrypsin | Sense: | | AAACACCCAGCCTACAACTAC | | 524 |
| | Antisense: | | GGACACGGAGCTGAGATTTATT | | |
| stbsdl | Sense: | | CACCAGGTAACCAAGGTCTATG | | 760 |
| | Antisense: | | CCCTGAGTATGTCGAGAATGAAG | | |
| stpla2g1b | Sense: | | AAATGCGATCCTTGGTCCTC | | 436 |
| | Antisense: | | CAACAGTAACGATCCCTGTCAA | | |

## Slide 2
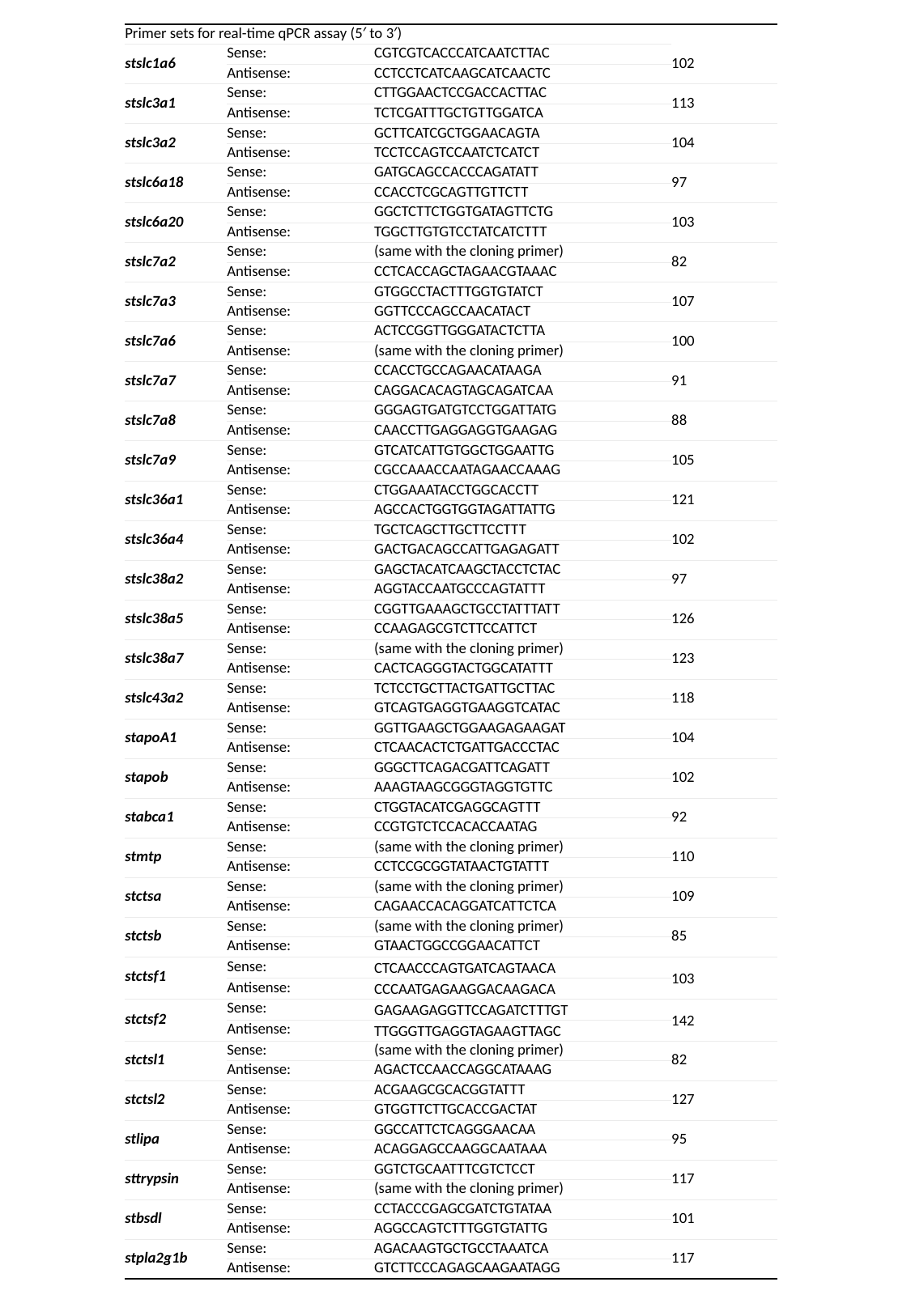

| Primer sets for real-time qPCR assay (5′ to 3′) | | | |
| --- | --- | --- | --- |
| stslc1a6 | Sense: | CGTCGTCACCCATCAATCTTAC | 102 |
| | Antisense: | CCTCCTCATCAAGCATCAACTC | |
| stslc3a1 | Sense: | CTTGGAACTCCGACCACTTAC | 113 |
| | Antisense: | TCTCGATTTGCTGTTGGATCA | |
| stslc3a2 | Sense: | GCTTCATCGCTGGAACAGTA | 104 |
| | Antisense: | TCCTCCAGTCCAATCTCATCT | |
| stslc6a18 | Sense: | GATGCAGCCACCCAGATATT | 97 |
| | Antisense: | CCACCTCGCAGTTGTTCTT | |
| stslc6a20 | Sense: | GGCTCTTCTGGTGATAGTTCTG | 103 |
| | Antisense: | TGGCTTGTGTCCTATCATCTTT | |
| stslc7a2 | Sense: | (same with the cloning primer) | 82 |
| | Antisense: | CCTCACCAGCTAGAACGTAAAC | |
| stslc7a3 | Sense: | GTGGCCTACTTTGGTGTATCT | 107 |
| | Antisense: | GGTTCCCAGCCAACATACT | |
| stslc7a6 | Sense: | ACTCCGGTTGGGATACTCTTA | 100 |
| | Antisense: | (same with the cloning primer) | |
| stslc7a7 | Sense: | CCACCTGCCAGAACATAAGA | 91 |
| | Antisense: | CAGGACACAGTAGCAGATCAA | |
| stslc7a8 | Sense: | GGGAGTGATGTCCTGGATTATG | 88 |
| | Antisense: | CAACCTTGAGGAGGTGAAGAG | |
| stslc7a9 | Sense: | GTCATCATTGTGGCTGGAATTG | 105 |
| | Antisense: | CGCCAAACCAATAGAACCAAAG | |
| stslc36a1 | Sense: | CTGGAAATACCTGGCACCTT | 121 |
| | Antisense: | AGCCACTGGTGGTAGATTATTG | |
| stslc36a4 | Sense: | TGCTCAGCTTGCTTCCTTT | 102 |
| | Antisense: | GACTGACAGCCATTGAGAGATT | |
| stslc38a2 | Sense: | GAGCTACATCAAGCTACCTCTAC | 97 |
| | Antisense: | AGGTACCAATGCCCAGTATTT | |
| stslc38a5 | Sense: | CGGTTGAAAGCTGCCTATTTATT | 126 |
| | Antisense: | CCAAGAGCGTCTTCCATTCT | |
| stslc38a7 | Sense: | (same with the cloning primer) | 123 |
| | Antisense: | CACTCAGGGTACTGGCATATTT | |
| stslc43a2 | Sense: | TCTCCTGCTTACTGATTGCTTAC | 118 |
| | Antisense: | GTCAGTGAGGTGAAGGTCATAC | |
| stapoA1 | Sense: | GGTTGAAGCTGGAAGAGAAGAT | 104 |
| | Antisense: | CTCAACACTCTGATTGACCCTAC | |
| stapob | Sense: | GGGCTTCAGACGATTCAGATT | 102 |
| | Antisense: | AAAGTAAGCGGGTAGGTGTTC | |
| stabca1 | Sense: | CTGGTACATCGAGGCAGTTT | 92 |
| | Antisense: | CCGTGTCTCCACACCAATAG | |
| stmtp | Sense: | (same with the cloning primer) | 110 |
| | Antisense: | CCTCCGCGGTATAACTGTATTT | |
| stctsa | Sense: | (same with the cloning primer) | 109 |
| | Antisense: | CAGAACCACAGGATCATTCTCA | |
| stctsb | Sense: | (same with the cloning primer) | 85 |
| | Antisense: | GTAACTGGCCGGAACATTCT | |
| stctsf1 | Sense: | CTCAACCCAGTGATCAGTAACA | 103 |
| | Antisense: | CCCAATGAGAAGGACAAGACA | |
| stctsf2 | Sense: | GAGAAGAGGTTCCAGATCTTTGT | 142 |
| | Antisense: | TTGGGTTGAGGTAGAAGTTAGC | |
| stctsl1 | Sense: | (same with the cloning primer) | 82 |
| | Antisense: | AGACTCCAACCAGGCATAAAG | |
| stctsl2 | Sense: | ACGAAGCGCACGGTATTT | 127 |
| | Antisense: | GTGGTTCTTGCACCGACTAT | |
| stlipa | Sense: | GGCCATTCTCAGGGAACAA | 95 |
| | Antisense: | ACAGGAGCCAAGGCAATAAA | |
| sttrypsin | Sense: | GGTCTGCAATTTCGTCTCCT | 117 |
| | Antisense: | (same with the cloning primer) | |
| stbsdl | Sense: | CCTACCCGAGCGATCTGTATAA | 101 |
| | Antisense: | AGGCCAGTCTTTGGTGTATTG | |
| stpla2g1b | Sense: | AGACAAGTGCTGCCTAAATCA | 117 |
| | Antisense: | GTCTTCCCAGAGCAAGAATAGG | |
