# Supplementary material for: Molecular mechanism of nutrient uptake in developing embryos of oviparous cloudy catshark (Scyliorhinus torazame)
Source: PLoS One. 2022 Mar 15;17(3):e0265428. doi: 10.1371/journal.pone.0265428 (PMC8923501; doi:10.1371/journal.pone.0265428)
Supplement: S2 Table — (PPTX) [file pone.0265428.s006.pptx]

## Slide 1
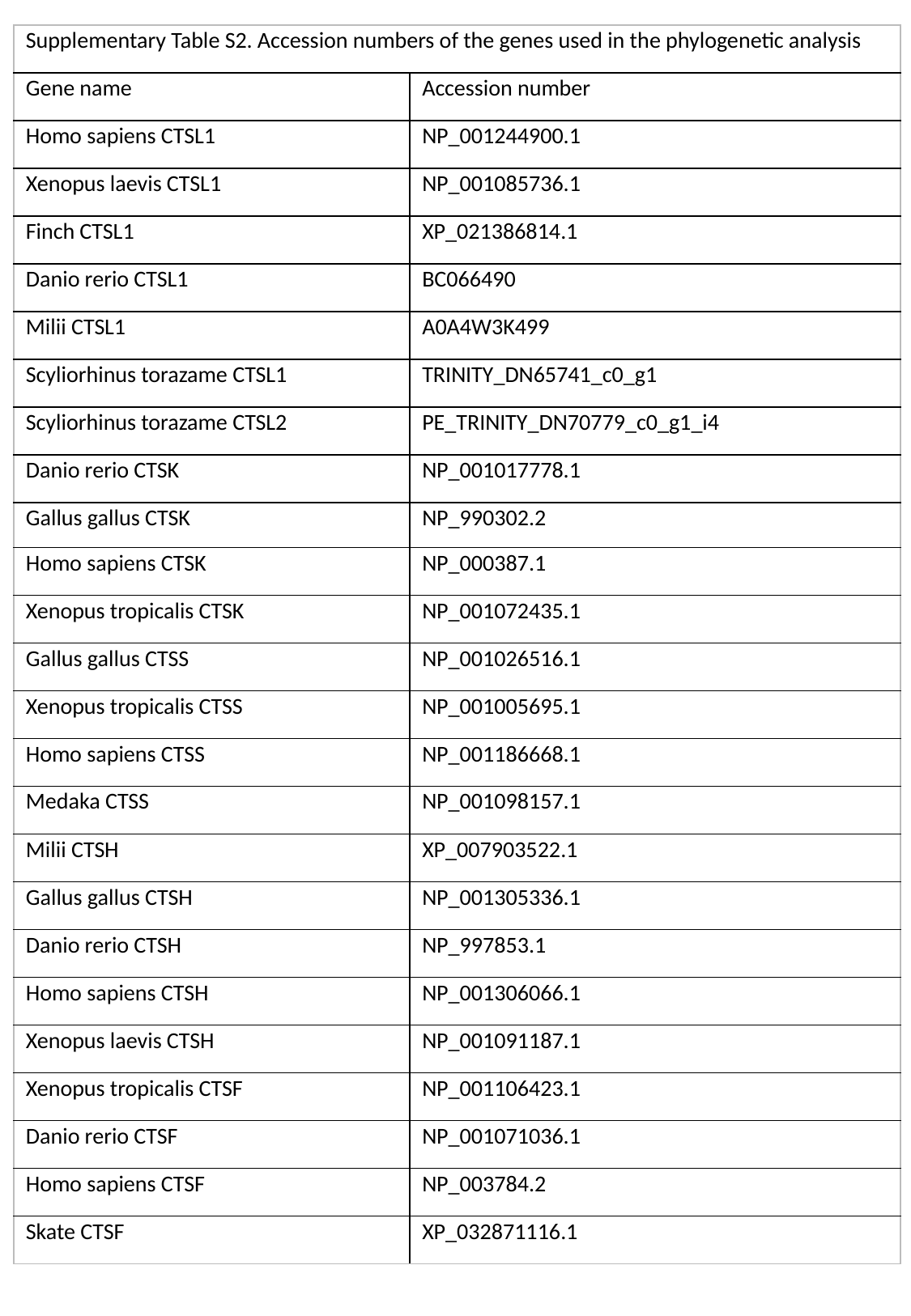

| Supplementary Table S2. Accession numbers of the genes used in the phylogenetic analysis | |
| --- | --- |
| Gene name | Accession number |
| Homo sapiens CTSL1 | NP\_001244900.1 |
| Xenopus laevis CTSL1 | NP\_001085736.1 |
| Finch CTSL1 | XP\_021386814.1 |
| Danio rerio CTSL1 | BC066490 |
| Milii CTSL1 | A0A4W3K499 |
| Scyliorhinus torazame CTSL1 | TRINITY\_DN65741\_c0\_g1 |
| Scyliorhinus torazame CTSL2 | PE\_TRINITY\_DN70779\_c0\_g1\_i4 |
| Danio rerio CTSK | NP\_001017778.1 |
| Gallus gallus CTSK | NP\_990302.2 |
| Homo sapiens CTSK | NP\_000387.1 |
| Xenopus tropicalis CTSK | NP\_001072435.1 |
| Gallus gallus CTSS | NP\_001026516.1 |
| Xenopus tropicalis CTSS | NP\_001005695.1 |
| Homo sapiens CTSS | NP\_001186668.1 |
| Medaka CTSS | NP\_001098157.1 |
| Milii CTSH | XP\_007903522.1 |
| Gallus gallus CTSH | NP\_001305336.1 |
| Danio rerio CTSH | NP\_997853.1 |
| Homo sapiens CTSH | NP\_001306066.1 |
| Xenopus laevis CTSH | NP\_001091187.1 |
| Xenopus tropicalis CTSF | NP\_001106423.1 |
| Danio rerio CTSF | NP\_001071036.1 |
| Homo sapiens CTSF | NP\_003784.2 |
| Skate CTSF | XP\_032871116.1 |
